# Anesthesia medication management competency in the post-anesthesia care unit: current challenges and strategies for enhancement—a qualitative study of nurses’ experiences

**DOI:** 10.3389/fmed.2026.1820937

**Published:** 2026-05-04

**Authors:** Renqiang Lu, Yuanfei Liu, Hongli Ma, Su Du, Ruyi Tan, Lin Lan, Chang Yang

**Affiliations:** Department of Anesthesiology, Chongqing University Cancer Hospital, Chongqing, China

**Keywords:** anesthesia medication, anesthesia nurses, competency development, PACU, qualitative study

## Abstract

**Objectives:**

With the continuous increase in surgical volume in China and the relative shortage of anesthesiologists, anesthesia nurses (ANs) play an increasingly critical role in perioperative care, particularly in the Post-Anesthesia Care Unit (PACU). Competency in anesthesia medication management is essential for patient safety during recovery. However, the training and professional development of ANs in China remain underdeveloped, with no standardized certification or continuing education systems in place.

**Aim:**

This study aimed to explore the current status, barriers, and potential pathways for improving anesthesia medication management competency among ANs in China.

**Methods:**

A qualitative descriptive design was employed, utilizing semi-structured interviews. Participants were selected via purposive sampling from a tertiary hospital in Chongqing between November and December 2025. Data were analyzed using Colaizzi’s seven-step phenomenological method with NVivo 12.0. Thematic analysis was conducted to identify key patterns and insights.

**Results:**

After analyzing interview content, three main themes and nine subthemes were distilled: (1) Cognition and Value Orientation of Anesthetic Medication Mastery—ANs recognized the necessity of medication knowledge for patient safety but exhibited gaps in comprehensive understanding, particularly regarding less frequently used drugs; (2) Multidimensional Barriers Impeding Mastery—including fragmented training mechanisms, limited learning motivation and resources, inadequate organizational and policy support, and deficiencies in departmental management; and (3) Targeted Demands for Enhancement—ANs expressed a need for systematic training, standardized assessment, role clarification, and interprofessional collaboration, especially with anesthesiologists.

**Conclusion:**

The study highlights a significant disparity between the recognized importance of anesthesia medication management and the actual competency levels among ANs. Barriers are systemic, rooted in training fragmentation, limited resources, and unclear professional roles. To enhance medication management capacity, a structured, policy-supported approach is recommended, including standardized training programs, role specialization, interdisciplinary collaboration, and incentive-linked professional development. These measures are essential for advancing anesthesia nursing as a specialized discipline and ensuring patient safety in PACU settings.

## 1 Introduction

The sustained growth in surgical volume in China, from 36 million procedures in 2011 to 88 million in 2023, has escalated the demand for perioperative anesthesia services (1). This demand starkly contrasts with the current density of anesthesiologists, which remains at 5.7 per 100,000 population, lower than the average of 6.89 in other upper-middle-income countries (2, 3). This workforce gap poses significant risks to patient safety and care quality during the perioperative period, where anesthesiologists are pivotal in providing anesthesia and monitoring vital signs (4, 5). To bridge this resource gap, the role of Anesthesia Nurses (ANs) has been introduced and expanded in China. Working under the supervision of anesthesiologists, ANs are tasked with providing nursing care during anesthesia, aiming to optimize the anesthesia workforce and enhance perioperative safety (6). The Post-anesthesia Care Unit (PACU) represents a critical phase where patients recover from anesthesia. This period is characterized by a high incidence of complications, necessitating vigilant monitoring and prompt intervention (7). Consequently, ANs are required to possess proficient competency in managing anesthesia-related medications—encompassing knowledge of indications, dosages, side effects, and the ability to recognize adverse drug reactions—to ensure timely identification of risks and safeguard patient recovery (8).

Despite this recognized need, the development of anesthesia nursing as a distinct specialty in China is still nascent (9). There is a lack of standardized definitions for ANs’ scopes of practice, particularly concerning medication management. Current guidelines primarily position ANs in a supportive role for handling crises, with no well-established national training, certification, or recertification systems dedicated to cultivating comprehensive medication management competencies (10). Existing literature on Chinese ANs has predominantly focused on general role perception or training needs assessments, with limited in-depth exploration of the current state of their anesthesia medication management competency, the multifaceted barriers they encounter in developing this competency, and their perceived pathways for improvement from a qualitative perspective (11, 12).

This gap in knowledge hinders the development of targeted strategies to strengthen this core competency. Therefore, utilizing a qualitative phenomenological approach, this study aims to explore the current status, barriers, and potential pathways for improving anesthesia medication management competency among ANs in China. Specifically, it seeks to: (1) describe their perceptions and the current state of this competency, (2) identify the barriers they face, and (3) elucidate their suggested pathways for enhancement. The findings are expected to provide empirical evidence to inform the formulation of standardized training and management frameworks for ANs in China, ultimately contributing to improved patient safety during the post-anesthesia recovery period.

## 2 Materials and methods

### 2.1 Design

This study employed a qualitative descriptive design, which is particularly suitable for exploring clinical nursing phenomena by providing a straightforward, comprehensive summary of participants’ experiences in everyday language, without imposing a highly interpretive or theoretical framework (13, 14). Semi-structured interviews were used to understand the current status of anesthetic drug management by ANs in the PACU, as well as the difficulties and needs encountered during their developmental process (15). For data analysis, we adopted Colaizzi’s seven−step phenomenological method (16), which allowed us to systematically extract meaning units and identify themes while preserving the essential structure of the lived experiences reported by participants. Combining a qualitative descriptive design with a phenomenological analytic approach enabled us to balance descriptive fidelity with interpretive depth. The study adhered to the Consolidates Criteria for Reporting Qualitative Research (COREQ) checklist (17).

### 2.2 Interview guide development

An initial interview guide was developed based on a review of domestic and international literature, consultations with one expert in anesthesiology and one in anesthesia nursing, and alignment with the research study aims. A pilot interview was conducted with three ANs of different professional titles, and the guide was subsequently refined according to the study’s purpose to form the final version (18). The final interview guide comprised the following questions: (1) What is your perspective on the necessity for anesthesia nurses to master anesthetic drugs? (2a) How would you evaluate your own level of mastery of anesthetic drugs? (2b) What channels or methods do you use to acquire knowledge related to anesthetic drugs? (3) In your opinion, what level of proficiency in anesthetic drug management should be achieved during the training of anesthesia nurses? (4) What difficulties have you encountered in the process of learning or being trained in anesthetic drug management? (5a) What additional support do you believe is needed for anesthesia nurses to learn about anesthetic drugs? (5b) What additional training do you believe is needed? (5c) What additional resources do you believe are needed? During the interview, the interviewer addressed each sub-question separately using probes to ensure comprehensive responses.

### 2.3 Participants

Based on the study aims, a purposive sampling method was adopted. The head nurse of the department provided the research team with a list of healthcare professionals who met the inclusion criteria. The researchers then directly invited these individuals to participate, explaining the voluntary nature of the study and assuring that non-participation or withdrawal would have no impact on their employment or relationship with the head nurse. To further minimize potential coercion, the head nurse was not informed of who ultimately agreed or declined to participate. Stakeholders involved in anesthetic drug management were recruited from the anesthesiology department of a tertiary hospital in Chongqing between November and December 2025 (19). Inclusion criteria were: (1) healthcare professionals engaged in or managing PACU work for 2 years or more; (2) for nurses, a valid Chinese nurse practice license; for the attending physician, a valid Chinese physician practice license (all licenses must be currently registered and in good standing); and (3) those who were willing and able to articulate their experiences and opinions clearly in Mandarin Chinese (the language of the interviews). Exclusion criteria were: (1) visiting nurses or physicians (i.e., those in temporary training or rotational positions); and (2) those absent from work for more than three months due to out-of-hospital training, maternity leave, or sick leave (applies to both nurses and physicians). The sample size (*n* = 7) aligns with recommended ranges for phenomenological studies (typically 5–10 participants), where saturation is achieved through interpretive depth rather than large numbers. Saturation was confirmed after the 7*^th^* interview, as no new themes emerged (20). General information of the study participants is presented in Table 1. All respondents provided informed consent and voluntarily participated in this study. Participants retained the right to refuse participation or withdraw from the interview at any time. This research was approved by the hospital’s Medical Ethics Committee.

**TABLE 1:** Characteristics of study participants (*n* = 7).

Code	Gender	Age	Education level	Professional title/position	Years of PACU work/management
N1	Female	38	Bachelor’s degree	Head Nurse/Associate Chief Nurse	15
N2	Female	56	Bachelor’s degree	Associate Chief Nurse	37
N3	Female	36	Bachelor’s degree	Nurse-in-Charge	5
N4	Male	28	Master’s degree	Nurse Practitioner	2
N5	Female	40	Bachelor’s degree	Nurse-in-Charge	3
N6	Female	38	Bachelor’s degree	Nurse Practitioner	4
N7	Female	43	Master’s degree	Attending Physician	20

### 2.4 Data collection

Prior to the study, the researchers contacted the leadership of the anesthesiology department to obtain support and permission. All data were kept confidential. Specifically, audio recordings and transcripts were stored on a password-protected computer accessible only to the research team. Participants were assigned codes (N1–N7), and all personal identifiers were removed from transcripts. No identifying information was included in any report or publication. Interviews were conducted by a Master of Nursing with specialized training in qualitative research methodology, who was not an AN. Neither the interviewer nor the note-taker had any prior working relationship with the participants or held any supervisory role over them. This independence was explicitly explained to participants before each interview to minimize any potential intimidation and to encourage open, honest sharing of their lived experiences. This background was intentionally chosen to minimize professional preconceptions and maintain a neutral stance during interviews. Another researcher was responsible for audio recording and note-taking; this second researcher also discreetly recorded key phrases (e.g., direct quotes, salient terms) and non-verbal expressions (e.g., pauses, sighs, posture changes) on a structured form. The interviews were conducted in a quiet office or a staff break room with only two researchers present: the interviewer and a second researcher responsible for audio recording and note-taking. Before each interview, the researchers explained the purpose and process of the study to the participants and assured them of the confidentiality of their personal information. The interviewer conducted each interview following the semi-structured interview guide described in section Participants, while listening attentively, and using non-judgmental prompts, avoiding any expression of personal opinions or reactions that might bias participant responses. When participants gave brief or ambiguous responses, the interviewer used non-directive probes such as: “Could you tell me more about that?”, “What did that experience feel like for you?”, “Can you give me a specific example?”, and “How did that situation affect your learning?” These probes encouraged participants to elaborate on their lived experiences without leading their answers. Each interview lasted 10–30 min. The variation in duration reflected differences in participants’ verbal expressiveness and availability; shorter interviews (10–15 min) contained concise but complete responses, and thematic saturation was confirmed across all interviews regardless of length.

### 2.5 Data analysis

After each interview, the interviewer and the note-taker compared their notes and observations to enrich data interpretation and capture both verbal and non-verbal cues. Within 24 h after each interview, one researcher transcribed the audio data verbatim into text and recorded non-verbal expressions in the corresponding sections of the document. NVivo 12.0 was used only for coding and theme management, not for transcription. To validate transcription accuracy, a second researcher independently compared a random 20% of the transcripts against the original audio recordings; any discrepancies were resolved through discussion. Two researchers independently analyzed, coded, and extracted themes from the data using NVivo 12.0 software following Colaizzi’s seven-step method. To implement bracketing (epoche), the interviewer maintained a reflective journal and the coders engaged in peer debriefing to suspend preconceptions. The two researchers were: (1) the interviewer (a Master of Nursing with training in qualitative methodology) and (2) another researcher experienced in qualitative analysis. After independent coding, they compared their identified themes and subthemes. Any discrepancies were resolved through discussion; if consensus could not be reached, the head nurse of the anesthesiology department was consulted to adjudicate. The specific analysis steps included: (1) transcribing the audio recordings into text to form a preliminary understanding; (2) identifying significant statements related to the research topic; (3) extracting and coding recurrent significant statements; (4) aggregating content with common characteristics to formulate themes; (5) integrating the research themes with participants’ statements to develop comprehensive descriptions; (6) articulating the essential structure of the phenomenon and refining it into final themes; and (7) verifying the accuracy and authenticity of the content with the participants. Upon completion of data analysis, the researchers returned the results to the participants for validation to ensure the accuracy of the information.

## 3 Findings

**FIGURE 1:**
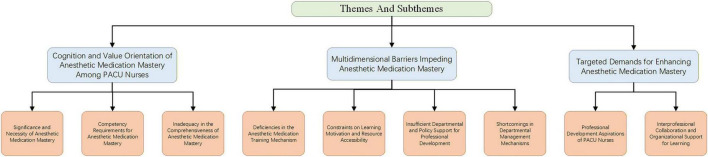
Themes and subthemes.

Following data collection and analysis, three main themes and nine subthemes were identified, revealing the cognitive perceptions, multidimensional barriers, and targeted demands regarding anesthetic medication management competency among PACU nurses. Figure 1 presents the three main themes and corresponding subthemes of this article.

### 3.1 Theme 1: cognition and value orientation of anesthetic medication mastery among ANs

#### 3.1.1 Subtheme 1: significance and necessity of anesthetic medication mastery

Participants universally emphasized that mastering knowledge related to anesthetic drugs—including indications, specifications, adverse effects, as well as the entire process management of dispensing, registration, recovery, and replenishment—is an indispensable core competency in anesthesia nursing practice. This understanding is closely associated with patient safety. As AN participate in the medical practice during patients’ anesthesia recovery period, their level of mastery of anesthetic drugs directly impacts the identification of adverse reactions and the assurance of medication safety.

“Since we are the ones directly caring for patients, we must have a thorough understanding of their conditions, including their reactions to medications and the indications for use. As nurses, these are things we should clearly understand, and even more so as specialist nurses. For patient safety and medication safety, these are all essential knowledge that must be mastered.” (N3)

Furthermore, anesthetic medication management is regarded as a specific core responsibility of ANs within the hospital operational system. Participants indicated that under the current hospital management model, the management of controlled anesthetic substances is primarily undertaken by ANs, constituting an integral and inescapable responsibility in their daily work.

“Judging from the operational model of many hospitals, the management of psychotropic substances and narcotic drugs actually still primarily relies on anesthesia nurses. This aspect of managing these drugs is therefore extremely important for anesthesia nurses.” (N4).

#### 3.1.2 Subtheme 2: competency requirements for anesthetic medication mastery

Participants unanimously emphasized that mastery of foundational knowledge regarding anesthetic medications is a fundamental competency requirement for AN.

“Managing patients in the PACU requires understanding the effects of medications and the key points for observation after administration—this is the most basic requirement” (N2).

This requirement is stratified according to clinical experience: junior nurses are expected to master knowledge of routinely used medications, while senior nurses—particularly core staff members—are required to systematically articulate pharmacological knowledge and assume teaching responsibilities.

“For ANs, we implement a stratified management approach. For junior nurses, they should at least know the content mentioned above. For senior nurses, especially our core nurses, they also undertake some teaching responsibilities. From my personal perspective, they should be able to briefly articulate the relevant pharmacological knowledge” (N2).

The timely identification of adverse drug reactions and the corresponding response are core competencies directly associated with patient safety. Participants emphasized that mastering the manifestations of adverse reactions to anesthetic medications enables early recognition of abnormal conditions during patients’ recovery period. This competency is regarded as a crucial safeguard for preventing complications during the recovery phase.

“Nurses must be able to observe adverse drug reactions and provide immediate feedback to the anesthesiologist for management” (N1).

Clinical collaboration and order verification abilities are regarded as critical practical competencies. Participants pointed out that solid medication knowledge serves as a prerequisite for effective collaboration with anesthesiologists.

“When anesthesiologists administer medications as prescribed, nurses need to be able to quickly prepare and accurately use the drugs” (N1).

“Medication knowledge is a necessary condition for nurses to identify potential problems in medical orders” (N2).

#### 3.1.3 Subtheme 3: inadequacy in the comprehensiveness of anesthetic medication mastery

Anesthesia nurses’ mastery of anesthetic medications is largely confined to routine clinical needs, with insufficient understanding of less frequently used medications and in-depth pharmacological details. The majority of nurses are only able to grasp the basic classifications of anesthetic drugs, lacking clear knowledge regarding the specific differences, advantages, and disadvantages of individual medications.

“I can roughly know which category a drug belongs to, but I’m not very clear about the specific differences between each drug, their advantages, disadvantages, and comparisons with others” (N6).

This limitation becomes more pronounced in the context of the operating room, with its diverse range of medications.

“There are actually many types of medications in the operating room, so the knowledge we master and are familiar with remains relatively fragmented and insufficiently comprehensive” (N4).

### 3.2 Theme 2: multidimensional barriers impeding anesthetic medication mastery

#### 3.2.1 Subtheme 1: deficiencies in the anesthetic medication training mechanism

The head nurse of the department noted that the clinical training system for anesthetic medications is relatively weak, and most nurses’ medication knowledge relies on accumulation through clinical practice.

“Knowledge is mostly acquired through clinical practice, with relatively few dedicated training programs, so competencies are naturally relatively insufficient” (N1).

A fragmented and unsystematic approach to training content represents the core issue within the training mechanism. Participants noted that routine learning of medication knowledge lacks organization, and training content was not structured according to drug categories or clinical scenarios.

“The medication knowledge they send out every day is very disorganized. Ideally, common medications should be categorized according to their usage categories” (N2).

This fragmented delivery of content results in poor learning outcomes.

“Content is simply divided up, lacking system, which leads to disorganized teaching and poor learning outcomes” (N2).

Shortcomings in training programs coupled with the absence of assessment and incentive mechanisms further exacerbate the inefficiency of the training system.

“Assessment is necessary—when there has been no assessment and no performance-based rewards or penalties, relying purely on self-motivation for learning is insufficient” (N1).

#### 3.2.2 Subtheme 2: constraints on learning motivation and resource accessibility

Participants indicated that the learning resources provided by the department lack refinement and synthesis, forcing them to passively consult drug inserts when encountering clinical problems, rather than relying on pre-integrated systematic materials.

“We need more refined and synthesized resources; relying on package inserts every time we encounter a problem is far from sufficient” (N1).

Heavy workload and time pressure further undermine learning capacity. ANs have broad responsibilities with unclear task delineation, and overtime work has become routine, leaving them with little energy for studying after work hours.

“The workload in the PACU is extremely heavy, and the division of responsibilities is unclear—we are tasked with too many duties. After working overtime every day, it’s difficult to find the energy to study during the limited rest time we have” (N6).

Meanwhile, the rotation requirements between anesthesia nursing and operating room nursing create a “dual learning burden,” further encroaching upon the limited time and energy available for study.

Insufficient intrinsic learning motivation exacerbates these difficulties. Nurses reported that specialized knowledge of anesthetic medications has limited practical application in daily work, as many complex medication-related procedures are independently performed by anesthesiologists.

“Learning about these psychotropic substances and narcotic drugs feels not very useful, because we rarely have opportunities for practical application” (N4).

“Many medication-related procedures in the operating room or PACU are independently performed by anesthesiologists, which diminishes our motivation to learn” (N1).

#### 3.2.3 Subtheme 3:insufficient departmental and policy support for professional development

Insufficient resource allocation and low priority given to anesthesia nursing by the department and hospital. From the perspective of both the department and the hospital, there is a lack of active support for training related to anesthetic medications, rooted in the perception that the work of ANs does not require in-depth medication knowledge.

“The hospital and department do not proactively encourage us to learn this, because they believe that AN’s work does not involve this area and there is no need to learn it” (N4).

Resources and training focus remain concentrated on operating room nursing, while investment in anesthesia nursing remains limited.

“The managerial resources, effort, and level of emphasis we invest in training are far less than those devoted to the subspecialty of operating room nursing—this is also a critical weakness of our discipline” (N1).

The development of anesthesia nursing as a specialty in China remains in its early stages and is constrained by policy factors. Some hospitals still employ the model of operating room nurses undertaking anesthesia nursing work, which cannot meet the developmental needs of the anesthesia nursing specialty.

“Our hospital still uses operating room nurses to perform anesthesia nursing work. This current situation cannot meet the developmental needs of the anesthesia specialty” (N2).

#### 3.2.4 Subtheme 4: shortcomings in departmental management mechanisms

Deficiencies in departmental management also negatively impacted ANs’ mastery of medications. Prominent issues included inadequate work management and role ambiguity, with nurses having broad responsibilities and insufficiently defined task delineation, which diverted attention from medication-related duties.

“We have a heavy workload with unclear division of responsibilities—we are tasked with too many duties, and the division of labor needs to be further refined” (N6).

Historical management legacies and outdated staffing models further constrain development. Some ANs resist nursing management due to ambiguous professional identity, and the excessive reliance on a single-point supervision model for personnel management is insufficient to support systematic specialized knowledge training.

“Some personnel identify more with anesthesia rather than nursing, and they are resistant to nursing management directives” (N1).

“When managers have limited energy, relying on a single individual for supervision is insufficient to support continuous specialized knowledge training” (N1).

The resolution of these structural problems requires policy adjustments at the institutional level.

### 3.3 Theme 3: targeted demands for enhancing anesthetic medication mastery

#### 3.3.1 Subtheme 1: professional development aspirations of ANs

Anesthesia nurses aspire to specialized career development and advocate for a “dedicated staffing” model in the PACU, where fixed positions would enable nurses to focus on their specific scope of responsibilities and cultivate more specialized medication management competencies.

“The PACU would be better managed by dedicated personnel, which would allow us more professional time to understand our assigned areas of responsibility and enhance our relevant competencies” (N5).

They also pointed out the limitations of self-study and fragmented knowledge, expressing a desire for senior clinical personnel or anesthesiologists to provide systematic, categorized instruction on anesthetic medications.

“Relying on self-study to learn pharmacological knowledge is quite difficult. If anesthesiologists could provide explanations in batches according to drug categories or commonly used medications, it would be more helpful for understanding” (N4).

Furthermore, they called for standardized training with integrated assessment mechanisms to ensure the systematic and effective nature of learning.

“We hope to receive systematic training and teaching with assessment mechanisms to enhance our abilities, allowing us to have a more systematic grasp of medications” (N5).

#### 3.3.2 Subtheme 2: interprofessional collaboration and organizational support for learning

Anesthesiologists, as professional practitioners of anesthetic medications, serve as pivotal contributors to specialized anesthesia medication training through their targeted instruction.

“Anesthesiologists sometimes give lectures to ANs, covering drug knowledge and theoretical content, which is very helpful” (N7).

Additionally, the department provides certain learning and development pathways, utilizing a combination of online and offline approaches to enhance nurses’ proficiency in medications.

The department has implemented a “Daily One Drug” sharing activity (N2).

The department also plans to reference national-level training courses, integrate them with clinical practice, and introduce expert-led curriculum resources to enhance teaching quality.

“We should learn from national-level training programs and integrate them into our local training to make learning more systematic” (N2).

## 4 Discussion

This study analyzed the cognition, barriers, and needs of ANs regarding anesthetic medication mastery through qualitative interviews. The findings not only reveal the practical dilemmas in current clinical practice but also provide empirical evidence to advance the specialized development of anesthesia nursing in China.

### 4.1 The discrepancy between perceived role and actual competency in anesthetic medication mastery among ANs

This study found that ANs generally recognize mastery of anesthetic medications as a core professional responsibility and a cornerstone of patient safety, which highly aligns with the emphasis on “anesthetic medication management competency” in China’s Training Syllabus for Nurses in Specialized Nursing Fields (21). However, the phenomenon of “selective mastery” of medication knowledge among nurses—characterized by familiarity with commonly used drugs but insufficient understanding of specialized medications and deeper pharmacological principles—reflects a significant discrepancy between perceived role and actual competency (22). The essence of this discrepancy lies in the transitional characteristics of China’s anesthesia nursing as it evolves from an “extension of operating room nursing” toward an “independent specialty.” On one hand, policy directives have clearly established the specialized status of ANs; on the other hand, clinical practice of ANs still relies on operating room nurses undertaking dual roles, resulting in a lack of systematic training in anesthetic medications and hindering the formation of comprehensive knowledge systems (23).

As evidenced by participants’ statements such as “medication knowledge mostly relies on clinical accumulation” and “insufficient understanding of non-routine medications,” this discrepancy is not a matter of individual capability but rather a direct consequence of systemic deficiencies in training. This finding aligns with conclusions from relevant domestic studies, indicating that the medication management competency of ANs in China currently falls short of meeting the demands of specialized development, necessitating a cognitive shift from “passive execution” to “active management” (10).

### 4.2 Underlying causes of multidimensional barriers: systematic contradictions in the transition toward specialization

The multidimensional barriers identified in this study—including deficiencies in training mechanisms, insufficient learning motivation, weak organizational support, and management shortcomings—are essentially a concentrated manifestation of the asynchrony between “policy advancement” and “clinical implementation” in the process of anesthesia nursing specialization in China.

#### 4.2.1 Fragmentation of the training system

The current training model for anesthetic medication among ANs primarily relies on “self-study and fragmented sharing,” lacking unified national curricula and assessment mechanisms. This situation is closely related to the late start of anesthesia nursing in China and the uneven regional development (24). Feedback from interviews—such as “disorganized training content” and “lack of systematic design”—confirms the reality that existing training is insufficient to support specialized capacity building. In contrast to mature systems in developed countries such as the United States and Australia, where ANs are required to complete accredited programs and pass specialized medication management assessments, China’s training system remains in an “exploratory stage,” urgently requiring the development of standardized, tiered training frameworks (25, 26).

#### 4.2.2 Structural deficiencies in organizational support

Issues such as insufficient departmental resource allocation and ambiguous scope of practice reflect the limitations of hospital management’s understanding of the value of the anesthesia specialty. Statements from interviews—such as “the hospital believes ANs do not need in-depth medication knowledge” and “nurses are excluded from intraoperative medication administration”—reveal that the traditional management mindset of “prioritizing surgery over recovery” remains dominant (27). This mindset not only restricts nurses’ clinical practice scenarios but also diminishes their learning motivation, creating a vicious cycle of “insufficient support → inadequate competency → low prioritization.” This predicament echoes the current reality under China’s Diagnosis-Related Groups (DRG) payment system, where “medical treatment is emphasized over nursing care,” highlighting the urgent need for coordinated breakthroughs in both policy and management to advance specialty development (28).

#### 4.2.3 Role ambiguity and professional identity challenges

The practice model of “operating room nurses concurrently undertaking anesthesia nursing work” has led to role ambiguity among nurses. Statements from interviews—such as “rotation systems hinder knowledge accumulation” and “identity conflicts”—reflect the mismatch between individuals and organizations during the specialization transition (29). This contradiction not only affects the continuous mastery of medication knowledge but also constrains nurses’ professional identity and developmental motivation, creating tension with China’s policy direction of “cultivating specialist nurses and enhancing the value of nursing.”

#### 4.2.4 Core responsibilities and pathways toward an independent specialty

Based on participants’ descriptions, the current core medication-related responsibilities of ANs include: dispensing and registering controlled substances, monitoring patients for adverse drug reactions, assisting anesthesiologists with medication tasks, and maintaining medication inventory. These tasks reflect a supportive rather than an independent role. To advance the specialty, we propose that future practice could incorporate advanced responsibilities such as: (a) independently administering select anesthetic drugs (e.g., reversal agents, sedatives, analgesics) under physician-approved protocols; (b) managing patient-controlled analgesia (PCA) and epidural infusions; (c) leading medication safety audits and quality improvement initiatives; and (d) participating in perioperative medication reconciliation. These expanded roles would leverage ANs’ full training potential, reduce anesthesiologists’ workload, and align with global trends in advanced practice nursing (e.g., CRNA scope in the US). Defining such a task profile is essential for developing targeted training curricula and competency assessment tools.

### 4.3 A demand-oriented development pathway: constructing a context-specific competency enhancement system for anesthetic medication management among ANs in China

Based on the findings of this study and considering the current developmental status of the anesthesia nursing specialty in China, the following targeted recommendations are proposed.

#### 4.3.1 Policy level: operationalizing the specialization framework

It is recommended to leverage national-level specialist nurse certification as an opportunity to clearly define the scope of practice and medication management responsibilities for ANs, incorporating anesthetic medication competency into core assessment indicators. Simultaneously, hospitals should be encouraged to establish additional anesthesia nursing-related items under the DRG payment system, utilizing policy leverage to enhance departmental prioritization and resource allocation for anesthesia nursing.

#### 4.3.2 Training level: constructing a standardized tiered training system

Drawing on the experience of national-level anesthesia specialist nurse training programs, develop specialized curricula for anesthetic medication management tailored to ANs. Implement a blended model combining “online theoretical learning + offline practical training + mentorship guidance,” integrated with formats such as “Daily One Drug” sharing activities and “case debriefings” to reinforce knowledge consolidation. Establish a tiered assessment mechanism featuring “foundation-building for junior nurses and advanced development for senior nurses,” using assessment as a driver for learning to address the problem of training fragmentation.

#### 4.3.3 Organizational level: optimizing interprofessional collaboration and management mechanisms

Promote the establishment of a “anesthesiologist-anesthesia nurse” collaboration mechanism, integrating anesthesiologists into the teaching team to participate in medication training and clinical supervision. Optimize anesthesia nurse position settings, gradually implementing “dedicated staffing for specialized management” to mitigate the impact of rotation on knowledge accumulation. Simultaneously, introduce information-based tools (such as smart medication cabinets and expiration date warning systems) to reduce nurses’ administrative burden, enabling them to focus on enhancing core medication management competencies.

#### 4.3.4 Individual level: stimulating intrinsic motivation for professional development

Strengthen nurses’ professional identity by establishing specialist role models and building academic exchange platforms. Establish a “competency-performance-promotion” linkage mechanism, integrating medication management abilities into professional title evaluation and performance-based allocation, thereby transforming the mindset from “passive learning” to “active learning” and fundamentally enhancing learning initiative.

## 5 Limitations and future directions

This study only selected a single-center sample, and the generalizability of the findings needs to be validated through multi-center studies. Future research could further conduct quantitative studies to develop an evaluation system for anesthetic medication management competency among ANs, and validate the effectiveness of enhancement strategies through interventional studies.

## 6 Conclusion

In summary, the current state of anesthetic medication mastery among ANs revealed in this study not only serves as a microcosm of the specialized development of nursing in China but also provides specific entry points for addressing dilemmas during the transitional period. Only through the synergistic efforts of policy guidance, system construction, and individual empowerment can the medication management competency of ANs be truly enhanced, promoting the evolution of anesthesia nursing in China from an “ancillary role” to a “core specialty,” ultimately ensuring patient safety and improving the quality of anesthesia nursing.

## Data Availability

The original contributions presented in this study are included in this article/supplementary materials, further inquiries can be directed to the corresponding author.
